# Dietary cadmium exposure from *Panax notoginseng*: cadmium accumulation and bioactive composition changes under cadmium stress

**DOI:** 10.3389/fnut.2026.1938789

**Published:** 2026-10-09

**Authors:** Mingyao Sun, Yunfei Li, Wanghai Cai, Tian Zheng, Meiling Chen, Luyan Zheng, Guiping Xu, Xia Zhou, Fei Miao

**Affiliations:** 1 Department of Clinical Nutrition, Shengli Clinical Medical College of Fujian Medical University, Fuzhou University Affiliated Provincial Hospital, Fujian Key Laboratory of Geriatrics Diseases, Fujian Provincial Center for Geriatrics, Fuzhou, China; 2 Shengli Clinical Medical College of Fujian Medical University, Fujian Provincial Hospital, Fuzhou, China; 3 Department of Obstetrics and Gynecology, Fuzhou First General Hospital Affiliated with Fujian Medical University, Fuzhou, China; 4 Department of Geriatric Medicine, Shengli Clinical Medical College of Fujian Medical University, Fuzhou University Affiliated Provincial Hospital, Fujian Key Laboratory of Geriatrics Diseases, Fujian Provincial Center for Geriatrics, Fuzhou, China

**Keywords:** cadmium, dietary exposure, flavonoids, food safety, meta-analysis, *Panax notoginseng*, risk assessment, saponins

## Abstract

**Background:**

*Panax notoginseng* is widely used as a dietary supplement for cardiovascular health, but cadmium (Cd) contamination may affect its safety and bioactive composition. A systematic assessment is lacking.

**Methods:**

We systematically searched Chinese and English databases for experimental studies on Cd residues and bioactive constituents in *P. notoginseng* under Cd exposure. Random-effects meta-analysis, subgroup analysis, random forest modeling, and consumer intake scenario estimation were performed.

**Results:**

Cd exposure increased Cd content by 184.17%, with larger increases under high-concentration (250.33%) and long-term (311.44%) exposure. Cd selectively reduced ginsenoside Re (−24.50%), total flavonoids (−24.19%), notoginsenoside R1 (−21.81%), ginsenoside Rd (−19.54%), and ginsenoside Rg1 (−16.05%). Calcium intervention was associated with a lower Cd accumulation response, decreasing the increase in Cd content from 356.67% to 44.22%, although this subgroup finding should be interpreted cautiously due to the limited number of available effect sizes. Under the predefined dietary exposure scenario with an intake of 6 g/day, contaminated *P. notoginseng* accounted for 6.8% of the provisional tolerable monthly intake (PTMI) for a 60 kg adult, increasing to 8.4% and 9.9% under the high-concentration and long-term exposure scenarios, respectively.

**Conclusion:**

Cd contamination may affect the quality and chemical safety evaluation of *P. notoginseng* by increasing Cd residues and altering bioactive constituent profiles. The dietary exposure analysis provides screening-level estimates of the potential contribution of *P. notoginseng*-derived Cd to dietary Cd intake under defined scenarios rather than a direct assessment of consumer health risk. Quality control should integrate monitoring of Cd residues, key bioactive constituents, processing-related Cd transfer, and actual intake patterns.

## 1 Introduction

The quality of plant-derived health foods and oral health products depends not only on the content and stability of characteristic bioactive constituents, but also on residual environmental contaminants, such as heavy metals, in the raw materials. *Panax notoginseng* is a perennial species of the genus *Panax* in the family Araliaceae. Its roots and rhizomes are the principal raw materials used in related oral preparations and health food products (1, 2). *P. notoginseng* is rich in dammarane-type saponins, mainly including notoginsenoside R1 and ginsenosides Rg1, Re, Rb1, and Rd, and also contains flavonoids, phenolic compounds, polysaccharides, amino acids, and other constituents (3, 4). These compounds possess distinct chemical structures and biological activities and have been associated with antioxidant, inflammation-modulating, and vasoprotective effects (5, 6). Accordingly, the contents of key saponins and flavonoids are important indicators of the chemical composition of *P. notoginseng* raw materials and are also relevant to the compositional consistency and quality stability of related products. Measurement of total saponins or a single marker compound alone may therefore be insufficient to capture broader compositional changes induced by environmental contamination.

Cadmium (Cd) is a toxic element with high mobility and bioaccumulation potential in soil–plant systems and is an environmental contaminant of particular concern in plant-derived foods and raw materials used in health products. Plant roots can absorb cadmium through divalent cation transport pathways and sequester it in the cell wall, apoplast, and vacuoles, while a proportion may also be translocated to aboveground tissues (7, 8). Pectin and hemicellulose in the root cell walls of *P. notoginseng* can participate in cadmium binding and immobilization, which may represent an important mechanism underlying cadmium accumulation in the roots (9). Because *P. notoginseng* is a perennial root medicinal plant whose harvested tissues remain in prolonged contact with the soil, cadmium concentrations in its roots are susceptible to sustained influences from soil conditions and the surrounding ecological environment (10). Cadmium present in *P. notoginseng* raw materials may be ingested directly in powdered products or partially transferred into finished products during extraction, concentration, and formulation, thereby contributing to consumers' overall cadmium intake. Cadmium is eliminated slowly from the human body, and long-term exposure may lead to its accumulation in the kidneys and other tissues, with chronic toxic effects including renal tubular dysfunction and abnormalities in bone metabolism (11, 12).

The effects of cadmium contamination are not limited to increased toxic element residues; cadmium may also alter the profile of bioactive constituents in *P. notoginseng*. Cadmium can induce the accumulation of reactive oxygen species, disrupt mineral nutrient uptake and cellular redox homeostasis, and alter carbon allocation toward secondary metabolic pathways (13, 14). Flavonoid biosynthesis involves key enzymes such as phenylalanine ammonia-lyase and chalcone synthase, whereas the formation of dammarane-type saponins depends on triterpenoid backbone biosynthesis, cytochrome P450-mediated hydroxylation, and glycosyltransferase-mediated glycosylation. Transcriptomic and genomic studies of *P. notoginseng* have identified multiple candidate genes involved in saponin biosynthesis and structural modification (15, 16), suggesting that cadmium-induced oxidative damage and metabolic reprogramming may further affect the formation of individual saponins. Cadmium contamination may therefore exert two interrelated but distinct effects: increasing the cadmium burden in raw materials and subsequent consumer intake, while selectively altering the composition of saponins and flavonoids and thereby reducing the compositional consistency of raw materials and finished products. It should be emphasized that a reduction in bioactive constituent levels does not directly imply diminished health effects or clinical efficacy in humans; nevertheless, it may serve as an important indicator of changes in product chemical composition and potential biological activity.

Previous studies have generally investigated either cadmium accumulation or changes in a limited number of bioactive constituents in *P. notoginseng* under specific cadmium concentrations, exposure durations, plant tissues, or mineral intervention conditions. For example, exogenous magnesium may reduce cadmium accumulation in *P. notoginseng* roots by regulating nitrate reductase-mediated nitric oxide production, whereas lime and oxalic acid treatments may simultaneously affect cadmium content, antioxidant defense systems, and the levels of saponins and flavonoids (17, 18). However, substantial heterogeneity exists across studies in terms of plant origin, cultivation conditions, cadmium treatment levels, exposure duration, analytical endpoints, and additional interventions. To date, a systematic quantitative evaluation integrating contaminant accumulation and bioactive constituent profiles within a unified framework remains lacking.

Accordingly, this study employed a systematic review and multilevel random-effects meta-analysis to quantify the overall effects of cadmium exposure on cadmium accumulation and on the major saponins, total saponins, and total flavonoids in *P. notoginseng*. Subgroup and moderator analyses were further conducted to evaluate the effects of exogenous cadmium level, exposure duration, plant part, and mineral interventions. On this basis, an exploratory random forest model with validation grouped by independent study was used to identify the principal predictors. In addition, a screening-level deterministic intake assessment was performed by integrating intake scenarios for *P. notoginseng*-related oral products with health-based guidance values for cadmium. This study aimed to elucidate the dual effects of cadmium exposure on contaminant safety and compositional quality in *P. notoginseng*-related health foods and oral botanical products, thereby providing quantitative evidence for source-area risk control, chemical quality evaluation, and consumer protection.

## 2 Data and methods

### 2.1 Literature search, eligibility criteria, and data extraction

This study systematically evaluated the effects of cadmium exposure on cadmium accumulation and the composition of major bioactive constituents in *Panax notoginseng*. The databases searched included PubMed, Web of Science, Embase, China National Knowledge Infrastructure (CNKI), Wanfang Data, and VIP Database. Each database was searched from inception to 17 May 2026, which was the final search date of this systematic review. The English search strategy was as follows: (“*Panax notoginseng*” OR Notoginseng OR Sanqi OR “San-qi” OR Sanchi) AND (cadmium OR Cd OR “heavy metal” OR “heavy metals” OR “metal stress”) AND (accumulation OR uptake OR residue OR toxicity OR stress OR exposure) AND (saponin OR ginsenoside OR notoginsenoside OR flavonoid OR “active constituent” OR “bioactive compound”). Database-specific search strategies were adapted according to the characteristics of each database, including controlled vocabulary terms where applicable and free-text keywords. The complete search strings used for all databases are provided in Supplementary Table S1. Two reviewers independently screened titles, abstracts, and full texts. Disagreements were resolved through discussion or, when necessary, consultation with a third reviewer. Study selection and reporting followed the PRISMA 2020 guidelines, and the detailed selection process is presented in Supplementary Figure S1. This systematic review was not prospectively registered in PROSPERO or any other public registry. However, the eligibility criteria, data extraction procedures, and statistical analysis framework were predefined before data synthesis and consistently applied throughout the review process.

Eligibility criteria were developed according to the PECO framework: (1) the study subjects were whole *P. notoginseng* plants or specific tissues, including roots, rhizomes, stems, and leaves; (2) the exposure consisted of experimental cadmium treatment, either alone or in combination with calcium, magnesium, or other mineral interventions; (3) a corresponding control group without cadmium treatment was included; (4) at least one outcome related to cadmium accumulation or bioactive constituents was reported, including cadmium content, total saponins, total flavonoids, notoginsenoside R1, or ginsenosides Rg1, Re, Rb1, and Rd; and (5) the mean, standard deviation or standard error, and sample size were available for both the treatment and control groups, or sufficient data could be extracted from figures or tables to calculate the effect size. Duplicate publications, studies without an appropriate control group, and studies lacking usable outcome data were excluded. When the same experimental dataset was reported in more than one publication, only the report containing the most complete information was retained.

Two reviewers independently extracted information on the first author, publication year, source of *P. notoginseng*, cultivation conditions, experimental system, plant part, exogenous Cd concentration, exposure duration, additional interventions, sample size, and the means and standard deviations of the treatment and control groups. Detailed study characteristics are provided in Supplementary Table S6. In total, 30 independent studies contributed 749 effect sizes, including 358 for Cd accumulation and 391 for bioactive constituent outcomes. Cd exposure concentrations ranged from 0.1 to 100 mg/kg, representing experimentally controlled stress conditions used to evaluate Cd accumulation and compositional responses in *P. notoginseng*. The numbers of independent studies and effect sizes contributing to individual outcomes are summarized in Supplementary Table S2.

Data reported only in graphical form were extracted using digitization software and independently verified by another reviewer. Because individual studies often reported multiple Cd concentrations, exposure durations, plant tissues, interventions, or outcome indicators, a single study could contribute multiple effect sizes. Accordingly, independent-study counts and effect-size counts were distinguished throughout data synthesis.

### 2.2 Effect size calculation and multilevel meta-analysis

The natural logarithm of the response ratio was used as the primary effect size to quantify the effects of cadmium (Cd) exposure on Cd accumulation and the composition of bioactive compounds in *Panax notoginseng* (Equation 1) (19, 20).


$$\begin{array}{l} \text{RR}=\text{In}\left(\frac{{\text{X}}_{\text{t}}}{{\text{X}}_{\text{c}}}\right)=\text{ln}\left({\text{X}}_{\text{t}}\right)\;-\;\text{ln}\left({\text{X}}_{\text{c}}\right) \end{array}$$ (1)

The sampling variance was calculated accordingly (Equations 2 and 3).


$$\begin{array}{l} \text{v}=\frac{\text{S}{{\text{D}}_{\text{t}}}^{2}}{{\text{n}}_{\text{t}}{{\text{X}}_{\text{t}}}^{2}}+\frac{\text{S}{{\text{D}}_{\text{c}}}^{2}}{{\text{n}}_{\text{c}}{{\text{X}}_{\text{c}}}^{2}} \end{array}$$ (2)

$$\begin{array}{l} w_{i}=\frac{1}{v_{i}+\tau^{2}} \end{array}$$ (3)

Here, (X_t_) and (X_c_) represent the mean values of the Cd-treated and control groups, respectively; (SD_t_) and (SD_c_) represent the standard deviations of the two groups; (n_t_) and (n_c_) represent the corresponding sample sizes; and (*v*_*i*_) denotes the variance of the effect size and τ^2^ denotes between-study variance.

When the original studies reported SD, these were converted to standard deviations using the corresponding formula (Equation 4).


$$\begin{array}{l} SD=\sqrt{n}\;\times \;SE \end{array}$$ (4)

To account for the non-independence of multiple effect sizes derived from the same study, such as those corresponding to different Cd concentrations, exposure durations, plant tissues, or outcome indicators, a three-level random-effects model was applied using study ID as the clustering unit. This approach was used to avoid underestimating standard errors due to the omission of correlations among effect sizes (Equation 5) (21, 22).


$$\begin{array}{l} y_{ij}=\theta +u_{j}+u_{i_{j}}+\varepsilon_{ij},u_{j}\sim N\;\left(0,\tau^{2}\right),\varepsilon_{ij}\sim N\;\left(0,v_{ij}\;\right) \end{array}$$ (5)

In the model, *y*_*ij*_ represents the *j-*th effect size from the *i-*th study; θ represents the overall mean effect; *u*_*i*_represents the between-study random effect, with [*u* ~ *N* (0, τ^2^)]; ε_ij_ represents the within-study random effect among effect sizes, wit [ε_*i*_~ *N*(0,*v*_*ij*_)] ;where *v*_*ij*_ was calculated using Equation 2.

Variance components were estimated using restricted maximum likelihood. Study ID was used as the clustering unit, and cluster-robust variance estimation with a small-sample correction was applied to calculate standard errors, 95% confidence intervals (CI), and the pooled effect size (RRw). Within-study dependency structures, such as those arising from shared control groups, were addressed using appropriate variance structures. Sensitivity analyses were also conducted by varying the assumed within-study correlation (Equations 6–8).


$$\begin{array}{l} {\text{RR}}_{\text{w}}=\frac{\sum_{\text{i}=1}^{\text{k}}{\text{w}}_{\text{i}}{\text{RR}}_{\text{i}}}{\sum_{\text{i}=1}^{\text{k}}{\text{w}}_{\text{i}}} \end{array}$$ (6)

$$\begin{array}{l} \text{SE}\left({\text{RR}}_{\text{w}}\right)=\sqrt{\frac{1}{\sum_{\text{i}=1}^{\text{k}}{\text{w}}_{\text{i}}}} \end{array}$$ (7)

$$\begin{array}{l} \text{95\% CI}={\text{RR}}_{\text{w}}\;\pm \;1.96\text{SE}\left({\text{RR}}_{\text{w}}\right) \end{array}$$ (8)

For ease of interpretation, the pooled RR was back-transformed into a percentage change (Equation 9).


$$\begin{array}{l} Percentage\;change\;\left(\%\right)=\left(e^{RRw}\;-\;1\right)\;\times \;100\% \end{array}$$ (9)

A percentage change greater than 0 indicated an increase in the Cd-treated group relative to the control group, whereas a value below 0 indicated a decrease. Between-study heterogeneity was assessed using the *Q*-test and the (*I*^2^) statistic (Supplementary Table S3). The appropriateness of subgroup classification was evaluated using the between-group heterogeneity test *Q*_*b*_, with (*P* < 0.05) indicating statistically significant differences among subgroups (23).

The Shapiro–Wilk test was also used to assess the distribution of effect sizes, which was visualized using a histogram fitted with a Gaussian distribution (24). The results indicated that the effect sizes deviated significantly from a normal distribution (*W* = 0.83; *P* < 0.001; Supplementary Figure S3).

Publication bias was assessed using Begg's test, Egger's test, Rosenthal's fail-safe number, and funnel plots when sufficient effect sizes were available (25–27). Because individual studies could contribute multiple dependent effect sizes, these assessments were considered exploratory evaluations of potential small-study effects rather than definitive evidence regarding the presence or absence of publication bias. Therefore, non-significant results from these tests were not interpreted as evidence that publication bias was absent. The *P*-values from both Begg's and Egger's tests were greater than 0.05, and the funnel plots showed relatively good symmetry, suggesting no obvious small-study effects were detected. However, these findings should be interpreted cautiously because within-study dependence among multiple effect sizes may influence conventional publication-bias diagnostics. In addition, the fail-safe numbers for all variables exceeded (5*N* + 10), suggesting that the overall results were relatively robust (Supplementary Table S2).

### 2.3 Subground and moderate analyses

To evaluate the moderating effects of Cd exposure conditions, plant tissues, and mineral interventions, subgroup analyses were conducted according to prespecified categories. External Cd concentrations were classified as low, medium, or high, as shown in Supplementary Table S4. Exposure duration was categorized as ≤ 60 days, 60–120 days, or >120 days. Plant tissues were classified as roots/rhizomes, stems, and leaves and were considered as a potential moderator in the Cd accumulation analysis. Tissue information was retained for each individual effect size during data extraction, and effect sizes from different plant tissues were not combined indiscriminately. This approach allowed tissue-specific differences in Cd accumulation to be evaluated and their potential contribution to heterogeneity to be assessed. Additional treatments were categorized as Cd exposure alone, calcium intervention, or magnesium intervention. Other interventions were summarized descriptively because of the limited number of available studies and were not included in the quantitative analysis.

For Cd accumulation outcomes, changes in the Cd content of *P. notoginseng* under different conditions were compared. For bioactive compound outcomes, the responses of total saponins, total flavonoids, notoginsenoside R1, and ginsenosides Rg1, Re, Rb1, and Rd were analyzed separately under the different conditions.

The effect size for each subgroup was estimated using a multilevel random-effects model and then back-transformed into a percentage change. Differences among subgroups were evaluated using an omnibus moderator test. A statistically significant effect within a subgroup does not necessarily indicate a significant difference between subgroups; only a significant moderator test provides evidence of a clear moderating effect. Findings from subgroups with a small number of studies were considered exploratory and interpreted cautiously.

### 2.4 Exploratory random forest analysis

To explore the major predictors associated with Cd accumulation and changes in bioactive compounds in *P. notoginseng*, separate random forest models were developed for Cd accumulation and bioactive compound outcomes. Random forest is an ensemble-learning method based on multiple decision trees and can be used to identify non-linear predictive relationships and complex interactions among variables (28, 29). In the Cd accumulation model, the response variable was the RR of Cd content in *P. notoginseng*. Candidate predictors included external Cd level, longitude, latitude, exposure duration, additional intervention, and plant tissue. Longitude and latitude were included as spatial proxy variables to capture geographical heterogeneity in the available dataset and were not considered direct measurements of specific soil, climatic, or other environmental characteristics.

In the bioactive compound model, the response variable was the RR of the corresponding compound content. Candidate predictors included additional intervention, external Cd level, longitude, latitude, exposure duration, and outcome type. Continuous variables were entered as numeric variables, whereas categorical variables were treated as factors. The importance of longitude and latitude was interpreted as predictive spatial information rather than direct evidence of specific environmental determinants.

To prevent data leakage caused by multiple effect sizes from the same study being simultaneously included in both the training and test sets, data partitioning was performed at the study level. All effect sizes derived from a given study were assigned exclusively to either the training set or the test set. Approximately 80% of the independent studies were used for model training, while the remaining 20% were used as an independent test set (30–32). Within the training set, repeated grouped cross-validation and grid search were used to optimize the number of trees, the number of variables considered at each split, and the minimum node size. Predictive performance in the independent test set was evaluated using the coefficient of determination (*R*^2^) and root mean square error (RMSE) (33). Variable importance was interpreted as the relative contribution of each predictor to model prediction rather than evidence of a causal effect on the outcome. Given the limited number of independent studies, the random forest analysis was considered exploratory and hypothesis-generating rather than a definitive predictive model.

The equations used to calculate RMSE and *R*^2^ are shown in Equations 10, 11, respectively.


$$\begin{array}{l} RMSE=\sqrt{\frac{1}{n}\overset{n}{\underset{i=1}{\sum }}{\left(y_{i}-{ŷ}_{i}\right)}^{2}} \end{array}$$ (10)

$$\begin{array}{l} R^{2}=1-\frac{\sum_{i=1}^{n}{\left(y_{i}-{ŷ}_{i}\right)}^{2}}{\sum_{i=1}^{n}{\left(y_{i}-ȳ\right)}^{2}} \end{array}$$ (11)

Here, *yi* represents the observed value, ŷ represents the predicted value, ȳ represents the mean of the observed values, and (*n*) represents the number of effect sizes in the test set.

### 2.5 Scenario-based estimation of consumer Cd intake and statistical analysis

To compare the relative contribution of Cd derived from *P. notoginseng* to dietary Cd exposure under different Cd exposure and mineral intervention scenarios, a screening-level deterministic intake scenario analysis was conducted. This approach was designed for comparative scenario evaluation rather than comprehensive dietary risk assessment, because processing-related Cd transformation, gastrointestinal bioaccessibility, absorption efficiency, background dietary Cd exposure, and individual consumption variability were not incorporated into the current model. Therefore, the estimated values represent scenario-based exposure indicators rather than actual absorbed Cd doses.

The roots and rhizomes of *P. notoginseng* are the principal raw materials used in *P. notoginseng* powder, extracts, and related oral products. Based on reported Cd residue levels in roots and rhizomes collected from major production areas in Yunnan Province, a concentration of 0.20 mg/kg was selected as a reference value for the baseline exposure scenario (34). This value was used only as an input parameter for comparative scenario analysis and should not be interpreted as a representative Cd concentration for all production areas, commercial batches, or *P. notoginseng*-containing products. It does not represent a regulatory threshold or a predicted Cd level in consumer products. The Cd concentration in *P. notoginseng* under each scenario was estimated accordingly (Equation 12).


$$\begin{array}{l} C_{Cd,scenario}=C_{Cd,baseline}\times \left(1+\frac{ES_{Cd}}{100}\right) \end{array}$$ (12)

Here, *C*_*Cd, scenario*_ represents the estimated Cd concentration in *P. notoginseng* under the corresponding scenario, expressed in mg/kg; *C*_*Cd, baseline*_ represents the baseline Cd concentration, set at 0.20 mg/kg; and *ES*_*Cd*_ represents the percentage change in Cd accumulation obtained from the overall meta-analysis or subgroup analysis.

Five scenarios were considered: a baseline scenario, an overall Cd exposure scenario, a high-concentration Cd exposure scenario, a long-term exposure scenario, and a calcium intervention scenario. The baseline scenario used only the concentration of 0.20 mg/kg, without applying an additional effect size. The other scenarios used the Cd accumulation effect sizes obtained from the overall analysis, the high-concentration subgroup, the long-term exposure subgroup, and the calcium intervention subgroup, respectively. The high-concentration and long-term exposure effects were derived from separate moderator analyses rather than from the same joint stratified model. Therefore, these effects were neither added nor multiplied but were compared as independent screening scenarios.

Because 1 mg/kg is numerically equivalent to 1 μg/g, daily Cd intake was calculated accordingly (Equation 13).


$$\begin{array}{l} EDI_{Cd}=C_{Cd,scenario}\times D_{PN}\times 1,000 \end{array}$$ (13)

Here, *EDI*_*Cd*_ represents the estimated daily intake of Cd derived from *P. notoginseng*, expressed in μg/day; *C*_*Cd, scenario*_ represents the Cd concentration in *P. notoginseng* under the corresponding scenario, expressed in mg/kg; and *D*_*PN*_ represents the daily intake of *P. notoginseng* raw material, expressed in g/day.

Based on the reported consumption range of 3–9 g/day for *P. notoginseng*, three intake scenarios of 3, 6, and 9 g/day were considered, with 6 g/day used as the primary comparison scenario (34). This range was used solely for the screening-level scenario analysis and does not represent the actual labeled recommended intake for all *P. notoginseng*-containing health foods or oral botanical products.

Monthly intake was calculated on the basis of 30 days, and an adult body weight of 60 kg was used in the primary analysis. The body-weight-adjusted monthly Cd intake was calculated accordingly (Equation 14).


$$\begin{array}{l} MI_{Cd,bw}=\frac{EDI_{Cd}\times 30}{BW} \end{array}$$ (14)

Here, *MI*_*Cd, bw*_ represents the body-weight-adjusted monthly Cd intake, expressed in μg/kg body weight/month, and BW represents body weight, which was set at 60 kg in the primary analysis.

The body-weight-adjusted monthly Cd intake was compared with the provisional tolerable monthly intake for Cd of 25 μg/kg body weight/month established by the Joint FAO/WHO Expert Committee on Food Additives (Equation 15) (35).


$$\begin{array}{l} \begin{array}{c} PTMIproportion\;=\frac{MI_{Cd,bw}}{25}\times 100\% \end{array} \end{array}$$ (15)

This proportion represents the incremental contribution of Cd derived from *P. notoginseng* to the tolerable Cd intake benchmark rather than the probability of Cd-related health effects. A value below the provisional tolerable monthly intake (PTMI) does not demonstrate the absence of safety concerns, because the PTMI reflects total Cd exposure from food, drinking water, and other sources rather than a safety threshold for a single food or product (36–38).

Multilevel meta-analysis, cluster-robust variance estimation, subgroup analysis, sensitivity analysis, and graphical visualization were performed in R version 4.3.3, primarily using the metafor and ggplot2 packages. Random forest models were developed using resampling procedures grouped by study ID, primarily with the ranger and caret packages. All statistical tests were two-sided, and (*P* < 0.05) was considered statistically significant. To ensure reproducibility, package versions, random seeds, and the main analysis code are provided in the Supplementary materials.

## 3 Results and discussion

### 3.1 Integrated effects of cadmium exposure on the chemical safety and bioactive-component profile of *Panax notoginseng*

The meta-analysis showed that cadmium exposure exerted two distinct effects on *Panax notoginseng*: it markedly increased the cadmium burden in plant tissues while selectively decreasing several key bioactive constituents (Figure 1). Cadmium exposure increased the cadmium content of *P. notoginseng* by 184.17% (*P* < 0.001), representing the largest relative change among all assessed outcomes. This finding indicates that *P. notoginseng* can absorb and accumulate substantial amounts of exogenous cadmium under experimental exposure conditions. As a perennial root plant, *P. notoginseng* remains in prolonged contact with the soil through its roots and rhizomes, which are also the principal raw materials used in *P. notoginseng* powder, extracts, and related oral products (39). Previous studies have shown that cadmium accumulation in *P. notoginseng* roots is closely associated with binding to root cell walls, the bioavailability of cadmium in soil, and cultivation conditions (9, 19, 33). Therefore, cadmium accumulation in the roots is relevant not only to production-area conditions and raw-material quality, but also to the chemical safety of finished products.

**Figure 1:**
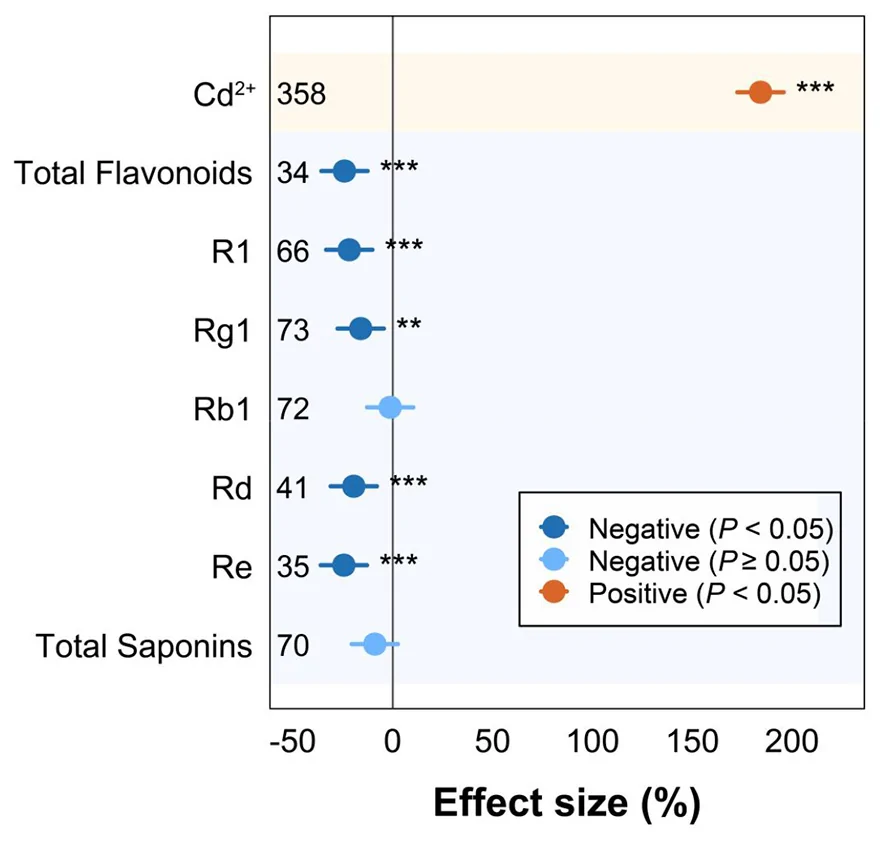
Overall effects of cadmium stress on cadmium accumulation and bioactive constituents in *Panax notoginseng*. Effect sizes are expressed as percentage changes relative to the control group. Orange indicates a significant positive effect, blue indicates a significant negative effect, and light blue indicates a non-significant negative effect. Numbers beside each outcome indicate the number of effect sizes included in the analysis. Error bars represent 95% confidence intervals. Asterisks indicate statistical significance: **P < 0.01 and ***P < 0.001. Cd^2+^ represents cadmium accumulation, while total flavonoids, R1, Rg1, Rb1, Rd, Re and total saponins represent pharmacological constituents of *Panax notoginseng*.

Cadmium is eliminated slowly after entering the human body and may accumulate over prolonged periods in the kidneys and other tissues. Long-term exposure has been associated with chronic toxic effects, including renal tubular injury and abnormalities in bone metabolism (37). However, the present study evaluated relative changes in cadmium concentrations in *P. notoginseng* tissues rather than the cadmium burden in consumers. Accordingly, the observed increase of 184.17% primarily reflects elevated contamination of the raw material. Its implications for human health depend on the cadmium concentration in finished products, transfer during processing, daily intake, consumption frequency, and concurrent cadmium exposure from other dietary and environmental sources.

Cadmium exposure also altered the bioactive-component profile of *P. notoginseng*. Ginsenoside Re showed the greatest reduction, decreasing by 24.50% relative to the control group (*P* < 0.001), followed by total flavonoids, which decreased by 24.19% (*P* < 0.001); notoginsenoside R1, by 21.81% (*P* < 0.001); ginsenoside Rd, by 19.54% (*P* < 0.001); and ginsenoside Rg1, by 16.05% (*P* < 0.01). By contrast, total saponins decreased by 8.99% and ginsenoside Rb1 by 1.29%. Although both showed downward trends, neither change reached statistical significance (*P* > 0.05; Figure 1).

These findings indicate that cadmium exposure does not cause all bioactive constituents in *P. notoginseng* to decline synchronously or proportionally. Instead, it appears to induce selective changes in the overall component profile. Notoginsenoside R1 and ginsenosides Rg1, Re, Rb1, and Rd are important marker compounds used to characterize the chemical composition of *P. notoginseng*. Saponin profiles vary substantially among tissues and growth stages, and individual saponins differ in both chemical structure and biological activity (40–42). Therefore, a non-significant change in total saponins does not indicate that all individual saponins remain stable. Differences in the direction and magnitude of changes among individual compounds may be partially offset in measurements of total content. Consequently, the use of total saponins or a single constituent as the sole quality-control indicator may fail to detect broader compositional changes induced by cadmium exposure.

The relatively large decreases in total flavonoids and ginsenoside Re indicate that these constituents showed greater sensitivity to cadmium exposure under the conditions evaluated in the included studies. Previous studies have suggested that flavonoid-related metabolism and saponin biosynthesis may respond to heavy-metal stress through oxidative stress and metabolic regulation. However, the present meta-analysis does not provide direct evidence regarding pathway regulation, enzyme activity, or gene expression changes. Therefore, these potential mechanisms should be considered as hypotheses requiring further experimental validation. Cadmium stress has been reported to induce oxidative stress responses and influence secondary metabolic processes in plants, including pathways associated with phenolic and flavonoid metabolism (43, 44). Previous studies suggest that flavonoid-related responses may participate in plant adaptation to metal stress; however, sustained oxidative damage may also disrupt flavonoid accumulation (44, 45). Similarly, changes in individual saponins may be associated with alterations in biosynthetic regulation, including hydroxylation and glycosylation processes involved in saponin formation (46, 47). However, the present meta-analysis did not directly assess gene expression, enzyme activity, or metabolic flux. Therefore, these biological processes should be regarded as potential explanations for the observed compositional changes rather than mechanisms demonstrated by the current study.

Notoginsenoside R1 is a representative characteristic constituent of *P. notoginseng*. Its reduction of more than 20% suggests that cadmium exposure may compromise the stability of a key chemical marker of the plant. Previous metabolomic and transcriptomic studies have suggested that saponin distribution among different tissues of *P. notoginseng* may be associated with tissue-specific regulation of biosynthetic processes (48). However, because the present study did not directly measure gene expression, enzyme activity, or metabolic flux, it cannot determine whether specific saponin biosynthetic pathways exhibit differential tolerance to cadmium stress. Nevertheless, the present study did not directly measure metabolic fluxes, key enzyme activities, or the expression of related genes. It therefore cannot be concluded that the protopanaxadiol-type saponin pathway is more tolerant to cadmium, nor can it be confirmed that cadmium exposure causes a redistribution of metabolic flux.

Overall, the effects of cadmium exposure on the quality of *P. notoginseng* are not limited to increased cadmium residues but also include alterations in the composition of key bioactive constituents. A reduction in constituent levels cannot be directly equated with diminished health-promoting effects or reduced biological responses in humans. Nevertheless, such changes may indicate reduced compositional consistency and altered potential bioactivity of the raw material. Quality evaluation of *P. notoginseng*-related oral products should therefore consider both cadmium residues and the multicomponent chemical profile rather than relying solely on a single cadmium limit or total saponin content.

### 3.2 Moderating effects of cadmium level, exposure duration, plant part, and mineral interventions

Subgroup analyses further showed that cadmium accumulation and changes in bioactive constituents in *Panax notoginseng* were associated with the level of exogenous cadmium, exposure duration, plant part, and additional mineral interventions (Figure 2). For cadmium accumulation, cadmium concentrations in *P. notoginseng* increased by 99.83%, 105.03%, and 250.33% in the low-, moderate-, and high-cadmium groups, respectively, and the effects within all subgroups were statistically significant (all *P* < 0.001; Figure 2a). The magnitude of change was substantially greater in the high-cadmium group than in the low- and moderate-cadmium groups, suggesting that a higher environmental cadmium burden may promote cadmium accumulation in *P. notoginseng* tissues. However, the effect estimates were similar between the low- and moderate-cadmium groups. Therefore, a strictly linear dose–response relationship should not be inferred unless supported by a statistically significant test of between-subgroup differences or continuous-dose meta-regression. Cadmium uptake by plants is also affected by soil pH, organic matter, cadmium speciation, cation-exchange capacity, coexisting ions, and root physiological status (49, 50).

**Figure 2:**
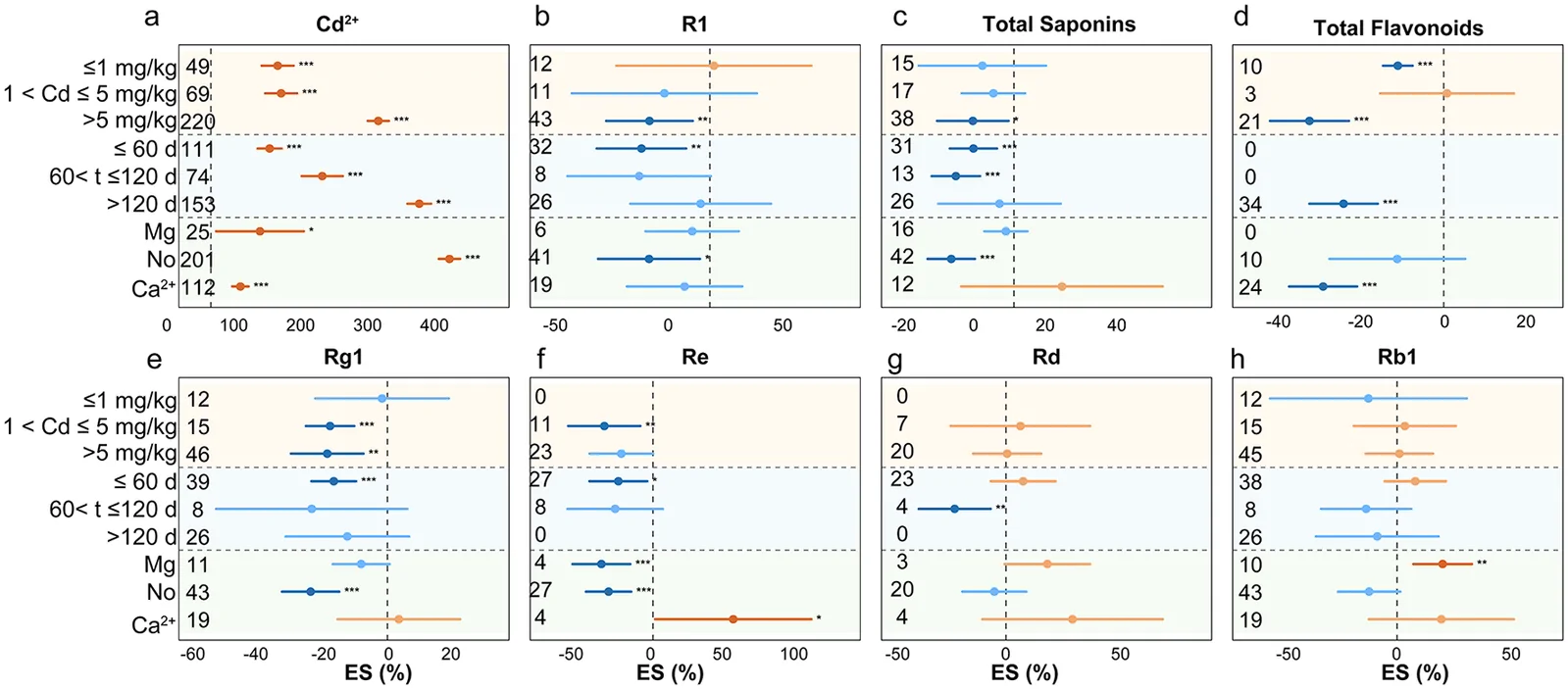
Subgroup associations between cadmium exposure conditions, intervention measures, and changes in cadmium accumulation and bioactive constituents in *Panax notoginseng*. Subgroup effect sizes are expressed as percentage changes relative to the corresponding control group. **(a)** Cd^2+^ accumulation; **(b–h)** changes in notoginsenoside R1, total saponins, total flavonoids, ginsenoside Rg1, ginsenoside Re, ginsenoside Rd, and ginsenoside Rb1, respectively. Error bars represent 95% confidence intervals. Numbers beside each subgroup indicate the number of effect sizes included. Orange points indicate significant positive effects; blue points indicate significant negative effects; light blue points indicate non-significant negative effects. Asterisks indicate statistical significance: *P < 0.05, ^**^P < 0.01, ***P < 0.001. Subgroups by low, medium, and high cadmium concentrations, exposure duration, and calcium or magnesium interventions were used to evaluate the modifying effects of exposure conditions and agronomic measures on chemical safety and bioactive composition.

Exposure-duration subgroups showed a clearer increasing pattern. Cadmium concentrations increased by 87.82%, 166.47%, and 311.44% in the short-, intermediate-, and long-term exposure groups, respectively, with statistically significant effects in all subgroups (all *P* < 0.001). These findings suggest that sustained exposure may facilitate the progressive accumulation of cadmium in *P. notoginseng* tissues. As a perennial root medicinal plant, *P. notoginseng* remains in continuous contact with the soil through its roots and rhizomes during cultivation. The background heavy-metal levels in cultivated soils and the intrinsic capacity of *P. notoginseng* to accumulate cadmium may therefore jointly affect cadmium residues in harvested raw materials (33, 51).

Subgroup analysis by plant part showed that cadmium concentrations increased by 300.72% in roots or rhizomes, 199.73% in stems, and 123.16% in leaves, with all *P*-values < 0.001 (Supplementary Figure S4). The magnitude of change followed the order roots or rhizomes > stems > leaves. These findings indicate that plant tissue type may represent an important source of biological heterogeneity in Cd accumulation responses, because different tissues reflect distinct processes of Cd uptake, transport, and storage within plants. After entering the plant through the roots, cadmium can bind to pectin, hemicellulose, and other components of the root cell wall or be compartmentalized in the root apoplast and vacuoles, thereby limiting its translocation to aboveground tissues (52, 53). Because roots and rhizomes are the principal raw materials used in *P. notoginseng*-related products and have a strong capacity to accumulate cadmium, control of root cadmium should be a priority in raw-material safety assessment (33).

Regarding mineral interventions, cadmium concentrations increased by 356.67% under cadmium exposure alone (*P* < 0.001), compared with increases of 44.22% in the calcium-intervention group (*P* < 0.001) and 73.45% in the magnesium-intervention group (*P* < 0.05). Both intervention subgroups showed smaller increases than the cadmium-only subgroup, with the smallest increase observed in the calcium group. However, because the subgroups may have been derived from different studies with varying cadmium concentrations, exposure durations, and cultivation conditions, these findings do not establish that calcium is more effective than magnesium in reducing cadmium accumulation.

Previous studies have shown that exogenous calcium may influence cadmium uptake and tissue distribution by increasing soil pH and regulating membrane-transport processes involving calcium-channel proteins, ATPases, and cation/H^+^ antiporters (54). Exogenous magnesium may reduce cadmium retention in the root cell wall of *P. notoginseng* by suppressing nitrate reductase-mediated nitric oxide production (17). In addition, lime application and foliar oxalic acid treatment may improve calcium nutrition and antioxidant defense, thereby alleviating cadmium toxicity in *P. notoginseng* (18). Potassium fertilization may also reduce the uptake of bioavailable cadmium by altering soil pH, organic matter, cation-exchange capacity, and microbial communities. Thus, different mineral elements may affect cadmium accumulation in *P. notoginseng* through distinct processes, including regulation of soil cadmium availability, root immobilization, transmembrane transport, and cellular redox status. Their mechanisms should not be simplified as direct competition among divalent ions.

The effects of mineral interventions on cadmium accumulation and bioactive-component profiles were not fully consistent. Notoginsenoside R1 decreased by 26.77% under high-cadmium conditions (*P* < 0.01), with the reduction occurring mainly during short- and intermediate-term exposure (Figure 2b), suggesting that R1 may be particularly sensitive to relatively high cadmium burdens. The biosynthesis of *P. notoginseng* saponins involves the mevalonate and 2-C-methyl-D-erythritol 4-phosphate pathways, which provide isoprenoid precursors, followed by squalene synthesis and epoxidation, oxidosqualene cyclization, CYP450-mediated hydroxylation, and UDP-glycosyltransferase-mediated glycosylation (15, 46, 55).

Total saponins increased by 13.67% in the calcium-intervention group (Figure 2c). However, an increase in total saponin content does not demonstrate that all individual saponins recovered to the same extent, because individual compounds may respond differently during biosynthesis, conversion, and accumulation. Total flavonoids showed a more consistently negative response to cadmium exposure. Their content decreased by 11.09% under low-cadmium conditions (*P* < 0.001), by 32.43% in the high-cadmium group (*P* < 0.001), by 24.19% in the long-term exposure group (*P* < 0.001), and by 29.14% in the calcium-intervention group (*P* < 0.001; Figure 2d). Cadmium exposure can induce reactive oxygen species accumulation and redox imbalance and substantially alter plant metabolic processes involving phenolic compounds, phenylpropanoids, and flavonoids (44, 45). These findings indicate that although the calcium-intervention subgroup exhibited a lower magnitude of cadmium accumulation, total flavonoid content was not maintained concurrently. Reducing the cadmium burden and preserving bioactive constituents are therefore related but non-interchangeable quality endpoints.

Individual saponins also exhibited component-specific responses. Ginsenoside Rg1 decreased by 17.87% and 18.75% under moderate- and high-cadmium conditions, respectively (*P* < 0.001 and *P* < 0.01), whereas it showed a numerical increase of 3.55% in the calcium-intervention group (Figure 2e). Ginsenoside Re increased by 54.68% in the calcium-intervention group (*P* < 0.05) but decreased by 35.05% in the magnesium-intervention group (*P* < 0.001; Figure 2f). Ginsenoside Rd decreased by 23.03% in the intermediate-duration subgroup (*P* < 0.01) and increased by 29.82% in the calcium-intervention group (Figure 2g). Ginsenoside Rb1 did not decrease significantly in any cadmium-concentration or exposure-duration subgroup. It increased by 16.08% and 16.54% in the calcium- and magnesium-intervention groups, respectively, with the increase reaching statistical significance in the magnesium group (*P* < 0.01; Figure 2h).

Large effect estimates, such as the 54.68% increase in ginsenoside Re in the calcium-intervention group, should nevertheless be interpreted cautiously in light of the number of independent studies, sample size, and heterogeneity within the corresponding subgroup. These findings suggest that protopanaxadiol- and protopanaxatriol-type saponins may respond differently to cadmium exposure and calcium or magnesium interventions. Such differences may be associated with structural characteristics of individual saponins, including aglycone structure and glycosylation patterns. However, the present evidence is insufficient to determine whether specific biosynthetic branches exhibit differential tolerance to cadmium stress (56, 57). However, the number of studies, exposure conditions, and comparison structures differed across subgroups, and the available evidence is insufficient to establish that any particular saponin biosynthetic branch is more tolerant to cadmium.

Overall, cadmium accumulation showed larger effect estimates under high-concentration and long-term exposure and in roots or rhizomes. Calcium- and magnesium-intervention subgroups exhibited relatively smaller increases in cadmium accumulation, but their effects on bioactive constituents were inconsistent. Some individual saponins were maintained or restored, whereas total flavonoids continued to decline. The effectiveness of agronomic interventions should therefore not be evaluated solely on the basis of cadmium residues; key individual saponins, total saponins, and total flavonoids should also be assessed concurrently.

### 3.3 Distinct predictive patterns for cadmium accumulation and changes in bioactive constituents

Random forest models were further used to identify the principal predictors associated with cadmium accumulation and changes in bioactive constituents in *Panax notoginseng* (Figures 3a, b). For the cadmium-accumulation model, the test-set (*R*^2^) was 0.75 and the RMSE was 0.56, indicating good predictive performance for the current test data. Variable-importance analysis showed that exogenous cadmium level had the highest relative importance (24.14%), followed by longitude (23.13%), latitude (23.08%), exposure duration (16.01%), additional intervention (9.85%), and plant part (3.78%; Figures 3a, b). The importance of exogenous cadmium level and exposure duration was broadly consistent with the subgroup-analysis results, indicating that contamination burden and exposure duration were major predictors of cadmium accumulation in *P. notoginseng* (Figures 3a, b).

**Figure 3:**
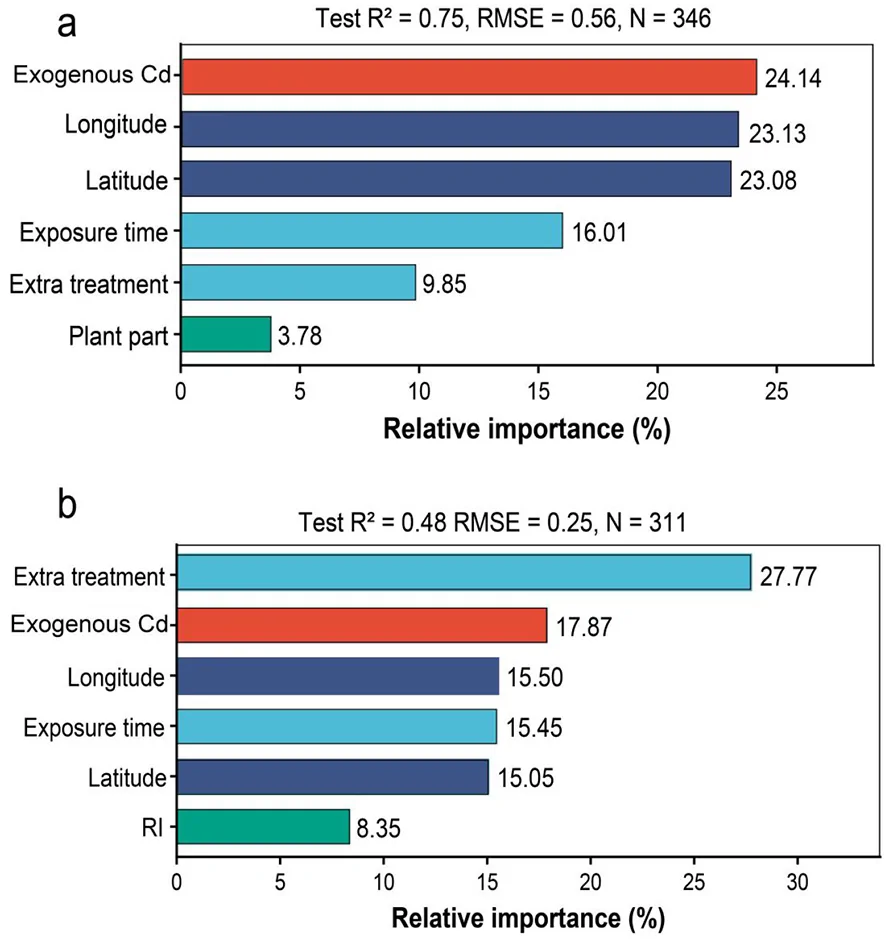
Random forest models for predicting cadmium residues and bioactive composition changes in *Panax notoginseng*. **(a)** Relative importance of predictors in the cadmium residue prediction model. **(b)** Relative importance of predictors in the bioactive constituent change prediction model. Numbers beside bars indicate relative importance (%). Model performance is shown above each panel as test-set *R*^2^, RMSE, and sample size (*n*). Exogenous Cd, external cadmium exposure level; Extra treatment, calcium, magnesium, or other interventions; RI, response indicator type (total saponins, total flavonoids, or individual saponins). Longitude and latitude were included as spatial proxy variables reflecting unmeasured geographical heterogeneity and should not be interpreted as direct indicators of specific soil, climatic, or cultivation characteristics.

It should be noted that variable importance in a random forest reflects the relative contribution of each predictor to model performance rather than a direct causal effect on the outcome. When predictors are correlated, importance may also be redistributed among correlated variables (58, 59). Although plant part showed relatively lower predictive importance compared with Cd level and exposure duration, tissue-specific differences remain biologically relevant because different plant tissues represent distinct processes of Cd absorption, translocation, and sequestration. Therefore, the relatively low importance of plant part should not be interpreted as evidence that tissue differences are biologically negligible. Instead, this finding may reflect the uneven distribution of data among plant tissues, correlations between plant part and other exposure-related variables, or the possibility that other predictors had already explained part of the variation associated with tissue-specific responses.

Longitude and latitude showed relatively high importance in the model but should not be regarded as direct causes of Cd accumulation. Rather, they should be interpreted as spatial proxy variables reflecting unmeasured geographical heterogeneity in the available dataset. Because soil pH, bioavailable Cd, organic matter, climatic conditions, cultivation practices, and rhizosphere characteristics were not consistently reported in the primary studies, the present model cannot determine which specific environmental factors underlie the observed spatial signal. Previous studies have shown that soil Cd contamination and rhizosphere conditions can influence Cd uptake and plant responses in *P. notoginseng* (60). In addition, geographical differences in the chemical profiles of *P. notoginseng* have been reported using chromatographic, spectroscopic, and metabolomic approaches (61–63). These previous findings provide biological context for the observed spatial heterogeneity but do not establish the specific environmental drivers represented by longitude or latitude in the present model. Accordingly, spatial variables may be useful for identifying broad geographical patterns and generating hypotheses regarding source-area heterogeneity, but they cannot replace direct measurements of total soil Cd, bioavailable Cd, or key soil physicochemical and climatic characteristics. Future source-area assessments should therefore combine geographical information with measured environmental variables before drawing conclusions regarding specific environmental drivers or production-area risk.

For the bioactive-constituent model, the test-set (*R*^2^) was 0.48 and the RMSE was 0.25, indicating lower predictive performance than that of the cadmium-accumulation model. Additional intervention had the highest relative importance (27.77%), followed by exogenous cadmium level (17.87%), longitude (15.50%), exposure duration (15.45%), latitude (15.05%), and outcome type (8.35%). These findings indicate that changes in bioactive constituents were not explained by exogenous Cd level alone. However, because plant developmental stage, light conditions, soil nutrients, and other environmental variables were not consistently available in the primary studies, their specific contributions could not be quantified in the present model.

Previous studies have shown substantial variation in saponin composition among different plant parts and production areas of *P. notoginseng*, with non-uniform distributions of protopanaxadiol- and protopanaxatriol-type saponins in roots, stems, and leaves (64). Moderate shading can influence saponin accumulation by regulating the expression of genes involved in saponin biosynthesis, and this effect may vary with plant age (65). Climatic factors, including temperature, precipitation, and humidity, are also closely associated with the contents of notoginsenoside R1 and ginsenosides Rg1, Rb1, and Rd, although individual saponins differ in the direction and magnitude of their responses to these factors (66). Environmental conditions may additionally regulate saponin accumulation through transcriptional modules associated with ginsenoside biosynthesis (67).

However, plant age, light exposure, temperature, soil nutrients, rhizosphere microorganisms, and the expression of key metabolic enzymes and regulatory genes were incompletely reported in most primary studies. These unmeasured factors may partly explain the lower predictive performance of the bioactive-constituent model.

The relative importance of outcome type was 8.35%, providing model-based support for response heterogeneity among individual saponins and total flavonoids. In addition to variation in saponin content, *P. notoginseng* from different production areas may show marked geographical differences in amino acids, organic acids, sugars, and other secondary metabolites (63). Studies based on multiwavelength chromatographic fingerprints have also shown that flavonoids, amino acids, and other compounds, in addition to the major saponins, can contribute to geographical discrimination and quality assessment of *P. notoginseng* (62). Consequently, measuring only total saponins or a single individual saponin may result in the loss of relevant information on changes in the broader component profile.

Overall, Cd accumulation and changes in bioactive constituents exhibited distinct predictive patterns. In the Cd-accumulation model, exogenous Cd level, longitude, latitude, and exposure duration showed relatively high predictive importance, whereas in the bioactive-constituent model, additional intervention had the highest importance, followed by exogenous Cd level, longitude, exposure duration, latitude, and outcome type. The importance of longitude and latitude should be interpreted as evidence of spatial predictive information rather than as identification of specific environmental determinants. These findings indicate that quality assessment of *P. notoginseng* products should incorporate two complementary endpoints: control of cadmium residues and evaluation of the bioactive-component profile.

The difference in model performance further suggests that systematic reporting of soil pH, total and bioavailable Cd, organic matter content, plant age, light and temperature conditions, mineral nutrition, rhizosphere microorganisms, and the expression of key metabolic genes may improve the predictive accuracy and external applicability of future models.

### 3.4 Effect-size-based scenario assessment of consumer cadmium intake

After confirming that cadmium exposure significantly increased cadmium accumulation in *Panax notoginseng*, a screening-level deterministic intake scenario analysis was conducted to compare the potential contribution of *P. notoginseng*-derived Cd to dietary Cd exposure under different predefined conditions. This analysis represents a screening-level assessment and cannot replace direct measurement of finished products or individualized health-risk assessments. Based on cadmium-residue surveys from major production areas, 0.20 mg/kg was used as the background scenario concentration. This value was applied only for comparisons among scenarios and does not represent a uniform cadmium concentration across products or a regulatory limit (68, 69). Under the predefined exposure scenarios, overall Cd exposure was estimated to increase the Cd concentration in *P. notoginseng* by 184.17% (0.568 mg/kg) relative to the baseline concentration. Under the high-Cd exposure, long-term exposure, and calcium-intervention scenarios, the estimated Cd concentrations were 250.33% (0.701 mg/kg), 311.44% (0.823 mg/kg), and 44.22% (0.288 mg/kg), respectively (Supplementary Table S5). These values represent scenario-based estimates derived from experimental responses and should not be interpreted as direct predictions of Cd concentrations in commercial *P. notoginseng* products. The high-Cd exposure and long-term exposure effects were derived from separate subgroup analyses and therefore were not combined as a joint scenario but were compared independently.

Using 6 g/day intake and a body weight of 60 kg as the primary scenario, the body-weight-adjusted monthly cadmium intake under the overall exposure scenario was 1.71 μg/kg bw/month, corresponding to 6.82% of the provisional tolerable monthly intake (PTMI; 25 μg/kg bw/month; Supplementary Table S5). The PTMI contribution rates under high-cadmium exposure, long-term exposure, and calcium-intervention scenarios were 8.41%, 9.87%, and 3.46%, respectively. Under the 3 g/day intake scenario, the corresponding contribution rates were 3.41%, 4.20%, 4.94%, and 1.73%; under the 9 g/day intake scenario, they were 10.23%, 12.61%, 14.81%, and 5.19%. None of the assessed scenarios exceeded the PTMI. However, the PTMI applies to total dietary cadmium exposure, and *P. notoginseng* represents only one potential source (Supplementary Table S5). Cereals, vegetables, seafood, and smoking can also contribute to cadmium exposure. Because cadmium has a long biological half-life and can accumulate in organs such as the kidneys, its contribution to cumulative body burden under long-term continuous intake should still be considered (69–71).

Notably, although calcium intervention reduced cadmium exposure in the scenario assessment, total flavonoid content still decreased by 29.14%, indicating that reducing contaminant burden does not necessarily result in simultaneous recovery of bioactive constituents (18). Therefore, evaluation of agronomic interventions should monitor both cadmium residues and key bioactive components rather than relying on a single contaminant-related endpoint. Quality evaluation of *P. notoginseng*-related oral products should integrate the entire chain from raw-material production to processing and consumption, incorporating production-area characteristics, raw-material residues, chemical composition, processing transfer rates, and actual intake levels into a unified framework (Figure 4).

**Figure 4:**
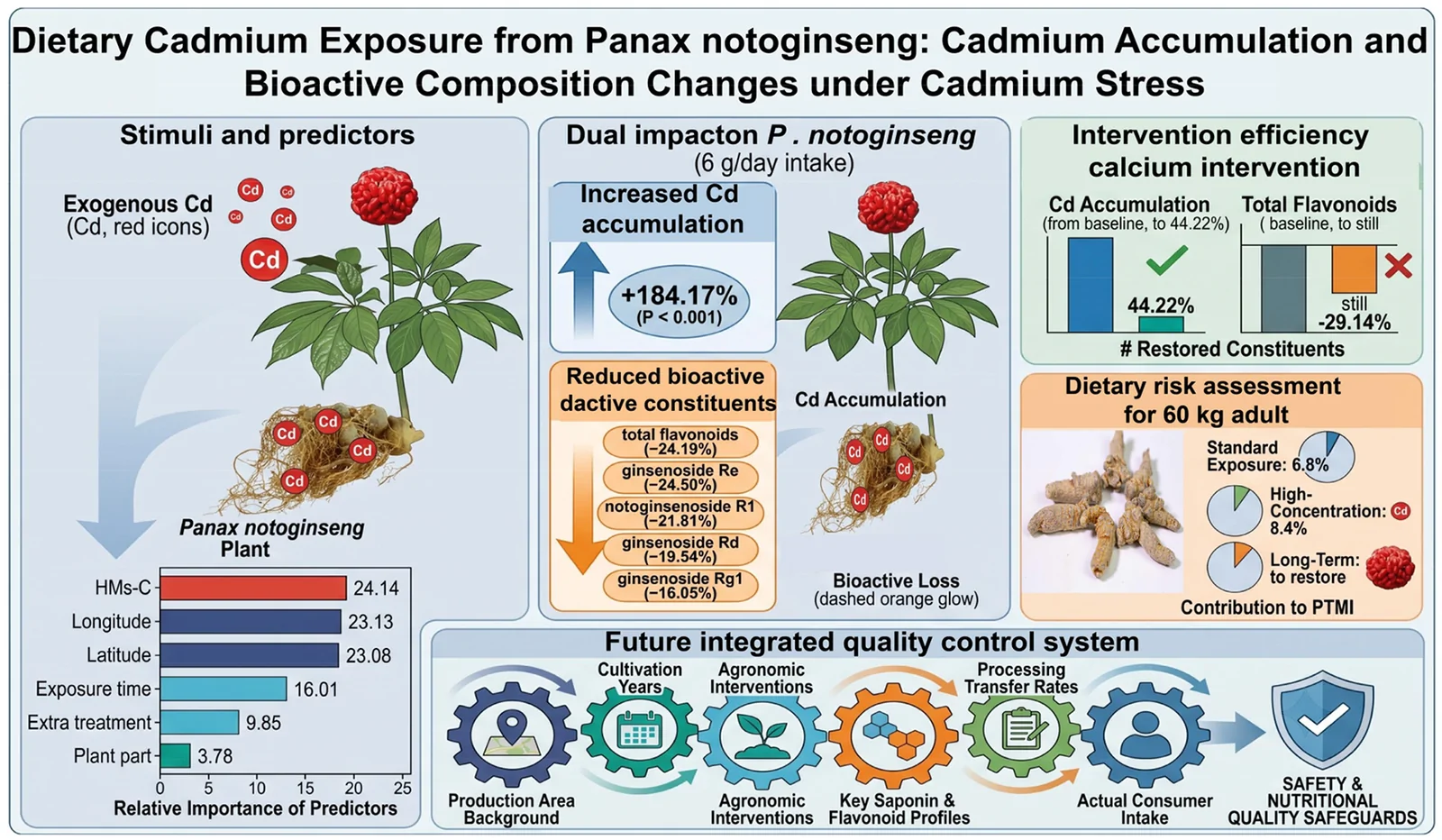
Proposed integrated linking cadmium accumulation, phytochemical alteration, dietary exposure, and quality control in *Panax notoginseng*.

### 3.5 Limitations and implications for chemical quality control of *Panax notoginseng* and related products

The findings of this study indicate that quality control of *P. notoginseng* and related oral products should move beyond conventional single-endpoint testing toward an integrated evaluation of cadmium residues and bioactive-component profiles. Control of cadmium contamination should begin at the cultivation stage by incorporating production-area soil conditions, irrigation-water quality, cultivation duration, and raw-material batch information into comprehensive quality management strategies (38, 53). Meanwhile, total saponin content alone may not adequately represent the overall chemical profile of *P. notoginseng*. Although the overall change in total saponins was not statistically significant in this study, notoginsenoside R1 and ginsenosides Re, Rg1, and Rd showed varying degrees of reduction. Recent studies have highlighted the complexity of plant biochemical responses to heavy-metal stress. Recent studies have highlighted the complexity of plant biochemical responses to heavy-metal stress. Toxic-metal exposure may influence phenolic and flavonoid accumulation, suggesting that secondary-metabolite responses are dependent on plant species and metal characteristics (72). In addition, heavy-metal accumulation should be interpreted together with exposure pathways and health-risk assessment parameters rather than solely based on tissue concentrations (73). Consistent with these findings, the present study considers Cd-induced changes in *P. notoginseng* bioactive constituents as stress-related responses, and the dietary exposure estimation as a screening-level scenario assessment rather than a direct prediction of consumer health risk. Therefore, when feasible, simultaneous determination of notoginsenoside R1 and ginsenosides Rg1, Re, Rb1, and Rd, together with total saponins and total flavonoids, is recommended for a more comprehensive evaluation of product compositional consistency (39).

Agronomic interventions should also be evaluated using dual endpoints of safety and quality. In the present study, calcium and magnesium treatments reduced cadmium accumulation in *P. notoginseng*, but total flavonoid content remained significantly decreased, suggesting that reducing contaminant levels does not necessarily preserve medicinal quality simultaneously (18). In addition, different product formulations may vary substantially in processing methods and concentration factors. Meta-analysis based on raw materials can only reflect overall trends in cadmium and bioactive-component changes and cannot replace direct measurement of cadmium levels and key constituents in finished products. Therefore, formulation-specific quality evaluations remain necessary for different types of oral products (74, 75).

Several limitations should also be acknowledged. First, the available evidence was primarily derived from controlled plant experiments, particularly artificial Cd-spiked pot studies, with limited information from naturally contaminated field soils, commercially available products, processing procedures, and real-world consumer exposure. The wide range of Cd concentrations used in the included studies reflects different experimentally imposed Cd stress intensities. However, these treatment concentrations should not be directly considered representative of Cd contamination levels commonly observed in agricultural soils, because Cd bioavailability and plant uptake under field conditions are influenced by soil physicochemical properties, Cd speciation, organic matter content, pH, and other environmental factors. Therefore, the observed Cd accumulation patterns should be interpreted cautiously when extrapolated to field production and commercial products. Second, substantial methodological heterogeneity existed among the included studies, including differences in *P. notoginseng* sources, cultivation conditions, Cd treatment concentrations, exposure durations, and analytical methods. Moreover, important environmental factors, such as soil Cd speciation, soil pH, organic matter content, and cultivation duration, were insufficiently reported in many studies (76, 77), which may represent major sources of residual heterogeneity. In addition, converting experimental Cd accumulation responses into dietary exposure scenarios introduces additional uncertainty because commercial *P. notoginseng* products may exhibit broader Cd concentration distributions than those represented by a single baseline value. Although sensitivity analysis using reported Cd concentration ranges was conducted to evaluate the influence of baseline concentration assumptions, the present exposure estimates should still be interpreted as comparative scenario-based assessments rather than direct predictions of Cd exposure from all commercial *P. notoginseng* products. Furthermore, publication-bias assessments should be interpreted cautiously because individual studies frequently contributed multiple dependent effect sizes. Although Begg's and Egger's tests were applied as exploratory evaluations of potential small-study effects, within-study dependence among effect sizes may influence conventional publication-bias diagnostics. Therefore, non-significant results from these tests should not be interpreted as definitive evidence that publication bias is absent.

Furthermore, this study evaluated changes in the chemical composition of *P. notoginseng* rather than direct alterations in pharmacological effects or clinical efficacy. The consumer exposure scenarios were estimated based on cadmium concentrations in raw *P. notoginseng* materials and did not account for cadmium migration, loss, or enrichment during washing, decoction, extraction, concentration, or formulation processes. Gastrointestinal bioaccessibility, human absorption efficiency, consumption frequency, and background cadmium exposure from other dietary sources were also not considered. Therefore, the intake scenarios presented here are suitable only for comparing the relative contribution of *P. notoginseng*-derived cadmium under different exposure conditions and should not be interpreted as actual absorbed doses or direct predictors of adverse health outcomes. Accordingly, the present results should not be considered a direct safety evaluation of commercial *P. notoginseng* products. In addition, the absence of probabilistic exposure modeling limits the ability to characterize population-level variability and uncertainty; therefore, future assessments should incorporate measured concentrations from commercial products together with probabilistic approaches.

Future studies should establish product databases covering diverse production areas, cultivation systems, raw-material batches, and oral formulations, with simultaneous monitoring of cadmium residues and key bioactive constituents. Processing studies should further characterize cadmium transfer behavior and bioactive-component retention during manufacturing, while in vitro digestion models could be applied to evaluate cadmium bioaccessibility. Consumer exposure assessments should ultimately be based on measured concentrations in commercially available products and incorporate probabilistic exposure modeling.

Overall, this meta-analysis demonstrated that cadmium exposure not only markedly increased cadmium accumulation in *Panax notoginseng* but also altered the composition of key bioactive constituents. Although calcium intervention effectively reduced cadmium accumulation, it did not fully restore phytochemical composition, indicating that reducing contaminant burden alone is insufficient to recover product quality. Deterministic dietary exposure assessment further suggested that *P. notoginseng*-derived cadmium contributed only a limited proportion of the PTMI under the evaluated scenarios; however, this contribution should be interpreted within the context of cumulative dietary cadmium exposure. Collectively, these findings indicate that cadmium contamination compromises both chemical safety and phytochemical quality through interconnected processes involving cadmium accumulation and secondary-metabolism disruption. As summarized in Figure 4, quality evaluation of *P. notoginseng* and related oral products should extend across the entire production-to-consumption chain. An integrated quality-control framework incorporating production-area characteristics, environmental and soil-risk management, cadmium residue monitoring, bioactive-component profiling, processing transfer, formulation characteristics, recommended intake levels, and consumer exposure would provide a more comprehensive assessment of the chemical safety and compositional consistency of *P. notoginseng* products.

## 4 Conclusion

Through meta-analysis, subgroup analysis, random forest modeling, and dietary exposure scenario estimation, this study systematically characterized the dual effects of cadmium exposure on *Panax notoginseng* as a dietary supplement. Cadmium exposure significantly increased the cadmium content of *P. notoginseng* by 184.17% and selectively reduced several key bioactive constituents, including ginsenoside Re (−24.50%), total flavonoids (−24.19%), notoginsenoside R1 (−21.81%), ginsenoside Rd (−19.54%), and ginsenoside Rg1 (−16.05%). Subgroup analyses and random forest modeling indicated that exogenous Cd level and exposure duration were important predictors of Cd accumulation, while longitude and latitude also showed relatively high predictive importance as spatial proxy variables. In the bioactive-constituent model, additional intervention showed the highest predictive importance. These predictive relationships should not be interpreted as direct evidence of specific environmental or metabolic mechanisms. Although calcium intervention was associated with a substantially lower Cd accumulation response (from 356.67% to 44.22%) in the available studies, this finding remains exploratory and requires further validation, it did not restore total flavonoid levels, which remained reduced by 29.14%. This finding indicates that lowering toxic-element residues does not necessarily preserve bioactive constituents. At a daily intake of 6 g, the screening-level exposure estimation indicated that *P. notoginseng*-derived Cd accounted for approximately 6.8% of the provisional tolerable monthly intake for cadmium in a 60-kg adult, increasing to 8.4% and 9.9% under the high-cadmium and long-term exposure scenarios, respectively. Overall, cadmium contamination may influence the chemical quality evaluation and safety management of *P. notoginseng* by increasing Cd residues in raw materials and altering bioactive-component profiles. These exposure estimates should be interpreted as comparative screening indicators under predefined scenarios rather than direct assessments of consumer health risk, because processing-related Cd transfer, gastrointestinal bioaccessibility, absorption efficiency, consumption variability, and background dietary Cd exposure were not incorporated. Future quality-control strategies should integrate measured production-area environmental characteristics, including soil Cd status and relevant physicochemical properties, together with cultivation duration, agronomic interventions, key saponin and flavonoid profiles, processing transfer rates, and actual intake levels.

## Data Availability

The original contributions presented in the study are included in the article/Supplementary material, further inquiries can be directed to the corresponding author/s.
