# Supplementary material for: Dietary cadmium exposure from Panax notoginseng: cadmium accumulation and bioactive composition changes under cadmium stress
Source: Front Nutr. 2026 Oct 9;13:1938789. doi: 10.3389/fnut.2026.1938789 (PMC13691592; doi:10.3389/fnut.2026.1938789)
Supplement: Supplementary file 1 [file Supplementary_file_1.docx]

Supplementary Material

**Figure. S1**: The PRISMA flow diagram. “n” is the number of studies.

**Figure. S2** Gaussian fitted histogram plotted using effect values (RR).

**Figure. S3.** Funnel plots for publication bias assessment of cadmium accumulation and secondary metabolite responses of Panax notoginseng under cadmium stress. The evaluated variables include cadmium content (Cd), ginsenoside Rb1 (Rb1), ginsenoside Rd (Rd), ginsenoside Re (Re), ginsenoside Rg1 (Rg1), ginsenoside Rf (Rf), total flavonoids, and total saponins. The x-axis represents the log-transformed response ratio, and the y-axis represents the standard error. The pink dashed line indicates the pooled effect estimate, while the orange dashed lines indicate the theoretical 95% confidence limits. Facet labels show the sample size (N), Begg test p-value, Egger test p-value, and trim-and-fill results when applicable. Triangles indicate imputed studies.

**Figure S4.** Subgroup analysis of Cd accumulation in different tissues of Panax notoginseng under cadmium exposure. Plant tissues were categorized as root, stem, and leaf. The x-axis represents the pooled effect size (ES, %), expressed as the percentage change in Cd content in the Cd-exposed group relative to the corresponding control group. Points represent the pooled effect estimates, and horizontal lines indicate the 95% confidence intervals. Numbers beside each tissue indicate the number of effect sizes included in the corresponding subgroup. Asterisks indicate the statistical significance of the pooled effect within each subgroup (*** *P* < 0.001). The magnitude of the pooled Cd accumulation response followed the order root > stem > leaf.

**Table S1. Database-specific search strategies used for the systematic literature search**

| Database | Platform | Search date | Search strategy |
| --- | --- | --- | --- |
| PubMed | PubMed | 17 May 2026 | ("Panax notoginseng" OR Notoginseng OR Sanqi OR "San-qi" OR Sanchi) AND (cadmium OR Cd OR "heavy metal" OR "heavy metals" OR "metal stress") AND (accumulation OR uptake OR residue OR toxicity OR stress OR exposure) AND (saponin OR ginsenoside OR notoginsenoside OR flavonoid OR "active constituent" OR "bioactive compound") |
| Web of Science | Web of Science Core Collection | 17 May 2026 | TS=("Panax notoginseng" OR Notoginseng OR Sanqi OR "San-qi" OR Sanchi) AND TS=(cadmium OR Cd OR "heavy metal" OR "heavy metals" OR "metal stress") AND TS=(accumulation OR uptake OR residue OR toxicity OR stress OR exposure) AND TS=(saponin OR ginsenoside OR notoginsenoside OR flavonoid OR "active constituent" OR "bioactive compound") |
| Embase | Embase | 17 May 2026 | ('panax notoginseng' OR notoginseng OR sanqi OR 'san qi' OR sanchi) AND (cadmium OR cd OR 'heavy metal' OR 'metal stress') AND (accumulation OR uptake OR residue OR toxicity OR stress OR exposure) AND (saponin OR ginsenoside OR notoginsenoside OR flavonoid OR 'bioactive compound') |
| CNKI | China National Knowledge Infrastructure | 17 May 2026 | (Panax notoginseng OR Notoginseng OR Sanqi) AND (cadmium OR Cd OR heavy metal OR metal stress) AND (accumulation OR uptake OR residue OR toxicity OR stress OR exposure) AND (saponin OR ginsenoside OR notoginsenoside OR flavonoid OR active constituent OR bioactive compound) |
| Wanfang Data | Wanfang Database | 17 May 2026 | (Panax notoginseng OR Notoginseng OR Sanqi) AND (cadmium OR Cd OR heavy metal) AND (accumulation OR uptake OR residue OR stress) AND (saponin OR ginsenoside OR flavonoid OR bioactive compound) |
| VIP Database | VIP Database | 17 May 2026 | (Panax notoginseng OR Notoginseng OR Sanqi) AND (cadmium OR Cd OR heavy metal OR metal stress) AND (accumulation OR uptake OR residue) AND (saponin OR ginsenoside OR flavonoid OR bioactive compound) |

**Table S2. Output results of Rosenthal fail-safe number test for different groups.**

| **Variable** | **Fail-safe number** | **Standard value** | **N** | **Result** |
| --- | --- | --- | --- | --- |
| Overall | 53,288,125.9 | 3755 | 749 | Robust |
| Re | 753.2 | 185 | 35 | Robust |
| Rd | 605.2 | 215 | 41 | Robust |
| Rb1 | 10,819.0 | 370 | 72 | Robust |
| Rg1 | 122,135.2 | 375 | 73 | Robust |
| R1 | 36,650.6 | 340 | 66 | Robust |
| Total saponins | 50,753.7 | 360 | 70 | Robust |
| Cd^2+^ | 44,339,305.1 | 1800 | 358 | Robust |
| Total Flavonoids | 923.1 | 180 | 34 | Robust |

**Table S3. Tests of between-group heterogeneity for each indicator across different grouping variables**

| **Variable** | **Subgroup** | **Q_B_** | ***P*** | **I²** |
| --- | --- | --- | --- | --- |
| Rb1 | Concentration | 2.3098 | 0.3151 | 13.41% |
|  | ExtraTreatment type | 19.3757 | < 0.0001 | 89.68% |
|  | Treatment duration | 41.6530 | < 0.0001 | 92.80% |
| Rg1 | Concentration | 11.3135 | 0.0035 | 82.32% |
|  | ExtraTreatment type | 71.4251 | < 0.0001 | 97.20% |
|  | Treatment duration | 0.7650 | 0.8578 | 3.21% |
| R1 | Concentration | 4.0261 | 0.1336 | 50.32% |
|  | ExtraTreatment type | 2.2157 | 0.3303 | 9.74% |
|  | Treatment duration | 9.5572 | 0.0227 | 68.61% |
| Total saponins | Concentration | 1.2515 | 0.5349 | 0.00% |
|  | ExtraTreatment type | 35.8156 | < 0.0001 | 94.42% |
|  | Treatment duration | 23.7731 | < 0.0001 | 87.38% |
| Cd^2+^ | Plant part | 14.6383 | 0.0007 | 86.34% |
|  | Concentration | 37.2360 | < 0.0001 | 94.63% |
|  | ExtraTreatment type | 154.8299 | < 0.0001 | 98.71% |
|  | Treatment duration | 3.7592 | 0.2887 | 20.20% |
| Rd | Concentration | 3.0227 | 0.2206 | 33.83% |
|  | ExtraTreatment type | 4.8641 | 0.0878 | 58.88% |
|  | Treatment duration | 10.6803 | 0.0136 | 71.91% |
| Total Flavonoids | Concentration | 11.9223 | 0.0026 | 83.22% |
|  | ExtraTreatment type | 1.9279 | 0.3814 | 3.12% |
|  | Treatment duration | 27.7232 | < 0.0001 | 89.18% |
| Re | Concentration/dose | 3.4997 | 0.0614 | 71.43% |
|  | ExtraTreatment type | 13.0208 | 0.0015 | 84.64% |
|  | Treatment duration | 13.4985 | 0.0002 | 92.59% |

**Note:** QB is the between-group heterogeneity statistic; *P* is the chi-square test probability value; I² was calculated as max[0, (QB - df) / QB] × 100%. *P* < 0.0001 was uniformly reported as < 0.0001.

**Statement:** According to the requirement that Biomass is the last variable, this table includes 13 identifiable indicators and 47 Between records up to Biomass. Content after Biomass was not included.

**Table S4.** Categorical variables and the subgroups within each variable

| Categorical variable | Subgroup | | |
| --- | --- | --- | --- |
| Plant part | Root | Stem | Leaf |
| Concentration | <1 mg/kg | 1-5 mg/kg | >5 mg/kg |
| Treatment type | NO | Ca^2+^ | Mg |
| Treatment duration | 0-60 days | 60 -120 days | >120 days |

**Table S5. Estimated Cadmium Exposure Scenarios for *Panax* *notoginseng* Based on Effect Sizes**

| **Exposure scenario** | **Estimated Cd residue (mg/kg)** | **Cd intake at 6 g/day (μg/day)** | **Monthly Cd intake (μg/month)** | **Body-weight-adjusted monthly intake (μg/kg bw/month)** | **PTMI proportion** |
| --- | --- | --- | --- | --- | --- |
| Baseline scenario | 0.20 | 1.20 | 36.0 | 0.60 | 2.4% |
| Overall Cd-stress effect size (+184.17%) | 0.57 | 3.41 | 102.3 | 1.71 | 6.8% |
| High-Cd exposure scenario (+250.33%) | 0.70 | 4.20 | 126.1 | 2.10 | 8.4% |
| Long-term exposure scenario (+311.44%) | 0.82 | 4.94 | 148.1 | 2.47 | 9.9% |
| Calcium intervention scenario (+44.22%) | 0.29 | 1.73 | 51.9 | 0.87 | 3.5% |

**Note.** PTMI = provisional tolerable monthly intake, calculated as 25 μg/kg bw/month. Estimates were calculated using a daily dose of 6 g *Panax notoginseng,* a reference adult body weight of 60 kg, and a 30-day month. Cd, cadmium; bw, body weight.

# Table S6. Characteristics of Included Studies

Characteristics of the independent studies included in the meta-analysis. The table summarizes experimental systems, Cd exposure characteristics, plant tissues, additional interventions, exposure durations, and outcome measurements.

| Study No. | First Author | Publication Year | Experimental System | Cd Concentration (mg/kg) | Exposure Duration (days) | Plant Tissue | Additional Intervention | Outcome Measurement | Number of Effect Sizes |
| --- | --- | --- | --- | --- | --- | --- | --- | --- | --- |
| 1 | Dongxu Li | 2020 | Pot experiment | 22.47 | 1 | Root | None | Cd accumulation | 30 |
| 2 | Zuran Li | 2021 | Pot experiment | 0.6; 6.0; 12.0 | 24 | Root; Stem | None; Calcium treatment | Cd accumulation | 72 |
| 3 | Pengfei Liu | 2021 | Pot experiment | 1.12 | 3 | Root | None; Magnesium treatment | Cd accumulation | 16 |
| 4 | Yanqun Zu | 2022 | Pot experiment | 0.6; 6.0; 12.0 | 240 | Root | None; Calcium treatment | Cd accumulation; Bioactive constituents | 75 |
| 5 | Guan-hua Cao | 2023 | Pot experiment | 2.62; 5.24; 10.48 | 150 | Root | Phosphorus treatment | Bioactive constituents | 12 |
| 6 | Chunyan Dai | 2025 | Pot experiment | 1.12; 5.0; 10.0; 15.0 | 30; 60 | Root; Stem | None; Magnesium treatment | Cd accumulation | 14 |
| 7 | Mingpeng Liu | 2025 | Pot experiment | 1.0; 10.0; 30.0; 60.0 | 120 | Root; Stem; Leaf | None | Cd accumulation; Bioactive constituents | 24 |
| 8 | Zuran Li | 2025 | Pot experiment | 0.6; 6.0 | 150 | Root; Stem | None; Polyethylene (PE) co-contamination | Cd accumulation | 24 |
| 9 | Mulan Wang | 2026 | Pot experiment | 0.5; 1.0; 1.5; 10.0; 20.0; 30.0; 40.0; 60.0; 80.0; 100.0 | 60; 75 | Root; Stem | None; Calcium treatment | Cd accumulation; Bioactive constituents | 23 |
| 10 | Lina | 2018 | Pot experiment | 0.6; 6.0; 12.0 | 40 | Root; Stem | Calcium treatment | Cd accumulation | 52 |
| 11 | Liujuan | 2020 | Pot experiment | 5.0; 10.0; 15.0 | 360 | Root; Leaf | None | Cd accumulation | 18 |
| 12 | Zhu Meilin | 2014 | Pot experiment | 0.1; 0.3; 0.6; 1.0; 3.0; 6.0; 10.0; 30.0 | 270 | Root; Stem; Leaf | None | Cd accumulation; Bioactive constituents | 56 |
| 13 | Li Suxia | 2020 | Pot experiment | 5.0; 10.0; 20.0; 30.0; 40.0; 50.0 | 60 | Root | None | Cd accumulation; Bioactive constituents | 30 |
| 14 | Shi Yue | 2019 | Pot experiment | 0.46; 0.92; 1.38 | 62 | Root | None; Potassium treatment | Bioactive constituents | 30 |
| 15 | Kan Qi | 2016 | Pot experiment | 2.5; 5.0; 10.0 | 1 | Root | None | Bioactive constituents | 19 |
| 16 | Li Dongxu | 2019 | Pot experiment | 2.0; 4.0; 6.0 | 1 | Root | Magnesium treatment | Bioactive constituents | 12 |
| 17 | Li Ziwei | 2018 | Field experiment | 5.0; 10.0; 30.0; 60.0 | 15; 30; 45; 60; 75 | Root; Stem; Leaf | None | Cd accumulation; Bioactive constituents | 48 |
| 18 | Jia Jieli | 2024 | Pot experiment | 10.0 | 60 | Root | Calcium treatment | Bioactive constituents | 12 |
| 19 | Qian jiaqi | 2021 | Pot experiment | 6;12 | 7;21 | Root | None | Bioactive constituents | 28 |
| 20 | Liao guyu | 2018 | Pot experiment | 10 | 3 | Root | None; Other treatment | Cd accumulation; Bioactive constituents | 16 |
| 21 | Xie fang | 2016 | Pot experiment | 10;15 | 12;30;120 | Root; Stem; Leaf | None | Cd accumulation | 36 |
| 22 | Luo cheng | 2025 | Field experiment | 21 | 10;25 | Root; Stem; Leaf | None; Other treatment | Cd accumulation | 12 |
| 23 | Mingpeng Liu | 2025 | Pot experiment | 5;10;20 | 0;15;30 | Root | None | Bioactive constituents | 6 |
| 24 | Mulan Wang | 2015 | Pot experiment | 10;20;30;40;50;60;70;80;90;100 | 7;14;21 | Root | Calcium treatment;None | Cd accumulation | 30 |
| 25 | Tianquan Lu | 2022 | Pot experiment | 1.4;5.6 | 21 | Root; Stem; Leaf | None | Cd accumulation | 20 |
| 26 | Li ziwei | 2018 | Pot experiment | 1;2;5 | 30;60 | Root | Calcium treatment | Bioactive constituents | 12 |
| 27 | Li haibo | 2014 | Pot experiment | 0.6; 1.2 ;4.8;6 | 40;120 | Root; Stem; Leaf | Other treatment | Cd accumulation; Bioactive constituents | 24 |
| 28 | Zhong ge | 2024 | Pot experimen | 2;5;10 | 14;30 | Root | None | Bioactive constituents | 12 |
| 30 | Li yiwei | 2019 | Pot experimen | 0.3;3;6;9 | 15;45 | Root; Stem; Leaf | Calcium treatment | Cd accumulation | 22 |
